# Determinants of Diabetic Nephropathy among Diabetic Patients in General Public Hospitals of Tigray, Ethiopia, 2018/19

**DOI:** 10.1155/2020/6396483

**Published:** 2020-09-21

**Authors:** Teklewoini Mariye Zemicheal, Degena Bahrey Tadesse, Hagos Tasew Atalay, Girmay Teklay Weldesamuel, Gebrewahd Bezabh Gebremichael, Haben Nuguse Tesfay, Teklehaimanot Gereziher Haile

**Affiliations:** ^1^Department of Adult Health Nursing, School of Nursing, College of Health Science and Comprehensive Specialized Hospital, Aksum University, Tigray, Ethiopia; ^2^Department of Pediatric Nursing, School of Nursing, College of Health Science and Comprehensive Specialized Hospital, Aksum University, Tigray, Ethiopia; ^3^Department of Adult Health Nursing, School of Nursing, College of Health Science and Comprehensive Specialized Hospital, Mekelle University, Mek'ele, Ethiopia; ^4^Department of Pediatrics, School of Medicine, College of Health Science and Comprehensive Specialized Hospital, Aksum University, Tigray, Ethiopia; ^5^Department of Maternity and Child Health Nursing, School of Nursing, College of Health Science and Comprehensive Specialized Hospital, Aksum University, Tigray, Ethiopia

## Abstract

**Background:**

Diabetic nephropathy is real damage resulting from having uncontrolled diabetes mellitus. Unmanaged diabetic nephropathy is one of the most leading causes of kidney failure. There is a scarcity of information on the determinants of diabetic nephropathy among diabetes mellitus patients in Ethiopia. Identification of the determinants can help devise a strategy to properly address the disease and its consequences. Therefore, this study was designed to assess the determinants of diabetic nephropathy among diabetes mellitus patients.

**Methods:**

Unmatched case-control study design with 168 cases and 672 controls with a mean age of 45.18 and 62.12, respectively, participated in the study. An interviewer-administered questionnaire was employed for data collection, and a systematic sampling technique was used to select the study participants. Data were entered into Epi data and exported to SPSS for data clarification and analysis. Binary logistic regression analysis was carried out to check the level of association between diabetic nephropathy and the independent variables.

**Results:**

Comorbidity (AOR: 4.96 at 95 CI: 1.77–13.87), hypertension (AOR: 6.33, 95% CI: 2.51–16.02), poor glycemic control (AOR: 3.27, 95% CI: 1.31, 8.21), age (AOR: 1.14, 95%: 1.09–1.19), duration with diabetes mellitus since diagnosis (AOR: 1.83, 95 CI: 1.62–2.06), and nonadherence to diabetic medication (AOR: 3.3, 95% CI: 1.34, 8.15), diet (AOR: 5.96, 95%: 1.92–18.54), and exercise (AOR: 5.60, 95% CI: 1.94–16.21) were the determinants of diabetic nephropathy.

**Conclusion:**

Adherence to medication, diet, and exercise should be empowered to achieve glycemic control and to prevent diabetic nephropathy. More attention has to be also given for old aged diabetic patients, long duration since diagnosis of diabetes mellitus, hypertension, and other comorbidities.

## 1. Introduction

Diabetic nephropathy (DN) is a clinical syndrome characterized by persistent microalbuminuria in concomitance with diabetes mellitus. It is diagnosed by the persistent increment of albumin or protein in urine when there is no other known renal disease [1, 2]. It is a microvascular complication of diabetes mellitus characterized by persistent proteinuria, decreased glomerular filtration rate, and increased blood pressure [3].

Diabetic nephropathy typically develops after diabetes duration of 10 years, or at least 5 years with type 1 diabetes but may be present at the time of diagnosis of type 2 diabetes [4]. If early prevention methods are not applied, diabetic patients with microalbuminuria typically progress to proteinuria and overt diabetic nephropathy. Approximately 20–40% of diabetic patients develop microalbuminuria within 10–15 years of the diagnosis of diabetes, and about 80–90% of those with microalbuminuria progress to more advanced stages. After 15–20 years, macroalbuminuria occurs in approximately 20–40% of patients, and around half of them present with renal insufficiency within 5 years [5]. Strikingly, 40–45% of patients with type 1 diabetes develop diabetic nephropathy and reach ESRD or die before its onset. Moreover, clinicians face a 30% prevalence of diabetic nephropathy among type 2 diabetic patients, with 45% of patients currently on dialysis having a primary diagnosis of diabetes [6].

The prevalence of diabetic nephropathy is still rising dramatically all over the world, with concomitant increases in associated mortality and cardiovascular complications [7]. Diabetic nephropathy is one of the most prevalent diabetes complications. With the global epidemic of diabetes mellitus, diabetic nephropathy has become an essential clinical and public health problem, with approximately one-third of them suffering from this long-term complication of diabetes [8]. It is a common and often devastating complication of diabetes mellitus associated with increased premature mortality and reduction in quality of life. It is a major factor in the development of chronic kidney disease and is the leading cause of end-stage renal disease [9] and the need for renal replacement therapy. It is also related to increased cardiovascular diseases and healthcare costs [10, 11].

The frequency of nephropathy in type 1 diabetes has a rather predictable prevalence (around 30–40%), whereas in type 2 diabetes, it depends on several factors [12]. The main risks of diabetic nephropathy are hyperglycemia and arterial hypertension, but the genetic susceptibility in both type 1 and type 2 diabetes is of great importance. Glomerular hyperfiltration, smoking, dyslipidemia, proteinuria, and dietary factors are also responsible for the development of diabetic nephropathy [13].

Screening for diabetic nephropathy should be done at least once a year, by assessing urinary albumin and estimated glomerular filtration rate in patients with type 1 diabetes mellitus of ≥5 years duration, in all type 2 diabetic patients, and in all diabetic patients with comorbid hypertension [4]. The initial treatment of diabetic nephropathy is prevention. Early detection of microalbuminuria and proper treatment may reverse or delay the progress of diabetic kidney disease. Patients should be treated to the lowest safe glucose level that can be obtained to prevent or control diabetic nephropathy. In addition to the aggressive treatment of elevated blood glucose, patients having diabetic nephropathy benefit from treatment with antihypertensive drugs [14].

DN was reported to be more common among diabetes patients in Africa than in the developed countries due to delayed diagnosis, insufficient screening and diagnostic services, poor control of blood sugar and other risk factors, and inadequate early stage care [6, 7]. Nonetheless, evidence supporting the burden of kidney disease in people with diabetes in Africa remains quite patchy and we are unaware of any attempts to synthesize existing data on the incidence of kidney disease in African diabetes populations.

Even though there are no clear definitions and there are different diagnostic criteria to assess the incidence and prevalence of diabetic nephropathy in Africa, it is a serious health threat for people with diabetes in the region with prevalence figures ranging from 11% to 83.7% [15]. The magnitude of diabetic complications in northern Africa ranges ranged from 6.7% to 46.3%. The magnitude of diabetic nephropathy in sub-Saharan Africa among patients with type 2 diabetes ranges 10% to 49% [16]. The prevalence of diabetic nephropathy in Ethiopia ranges from 2% to 30% with an average of 15% [17].

In general, it has been found that despite preventive and therapeutic managements being available for diabetic nephropathy, a significant number of patients develop diabetic nephropathy. There is limited information on the burden of chronic kidney disease among diabetes mellitus patients in sub-Saharan Africa. Thus to prevent its devastating consequence, it is imperative to identify the determinants of diabetic nephropathy so that public health, preventive measures could be in place to decrease the morbidity and mortality of diabetic nephropathy. Hence, this study is aimed to assess the determinants of diabetic nephropathy among diabetic patients in general public hospitals of Tigray, Ethiopia.

## 2. Methods

### 2.1. Study Area and Period

Tigray is one of the nine regions of Ethiopia. The region has a total of 5,247,005 population with 2,660,002 females and 2,587,003 males [18]. The study was conducted in public hospitals of Tigray in which there are a total of fourteen general and two referral public hospitals. The study was conducted from September 1 to December 30, 2018, G.C.

### 2.2. Study Design

A hospital-based unmatched case-control study was conducted.

### 2.3. Source Population

  Cases: patients with diabetic nephropathy in general public hospitals of Tigray at the time of data collection
  Controls: Diabetic patients without diabetic nephropathy in general public hospitals of Tigray at the time of data collection

### 2.4. Study Population

  Cases: all the selected diabetic patients with diabetic nephropathy in the general public hospitals of Tigray
  Controls: all the selected diabetic patients without diabetic nephropathy in the general public hospitals of Tigray

### 2.5. Eligibility Criteria

#### 2.5.1. Inclusion Criteria

  Cases: all diabetic patients with diabetic nephropathy were included as cases
  Controls: all diabetic patients without diabetic nephropathy were included as controls, respectively

#### 2.5.2. Exclusion Criteria

Critically ill patients and pregnant mothers (gestational DM) were excluded from the study.

### 2.6. Sample Size Calculation

Epi Info software version 7.1.1 was used to calculate sample size considering the following parameters. Significance = 95%; Power = 80%; Odds ratio = 2.13, which is the odds of diabetic nephropathy among diabetic patients with hypertension taken from a study conducted in Ayder Comprehensive Specialized Hospital, Tigray, Ethiopia, case to control ratio = 1 : 4, proportion of controls with exposure = 24.62%, and proportion of cases with exposure = 41% [19]. The total sample size was 420 with 84 cases and 336 controls. Considering a design effect of 2, the overall sample taken was 840 with 168 cases and 672 controls.

### 2.7. Sampling Technique and Procedure

Of the 14 general public hospitals in the regional state of Tigray, six hospitals were selected using the lottery method, and the calculated sample size was allocated proportionally to the selected hospitals based on the number of patients attending at the hospitals. The study subjects were selected using a systematic sampling technique every *K*^th^ interval. *K*^th^ interval was calculated by dividing the total number of cases and controls (N) by the total sample size (*n*) of cases and controls.

### 2.8. Study Variables

(i) Dependent variable
  • Diabetic nephropathy
(ii) Independent variables
• Sociodemographic factors: sex, age, education status, residence, marital status, occupation, ethnicity, and religion; clinical characters: BMI status, duration with DM since diagnosis, hypertension, comorbidity, glycemic control, and behavioral factors (diabetic diet adherence, exercise adherence, medication adherence, alcohol intake, and smoking)

### 2.9. Data Collection Tool

A structured questionnaire was used for data collection. It had three parts. Part I: social demographic data; Part II: Respondents' health profile; Part III: Summary of Diabetic Care Activity (SDCA), which analyzes the self-practice domains of diabetes such as diet, exercise, smoking, and alcohol consumption. The reliability of the SDCA questionnaire was tested on similar studies conducted in Ethiopia [20, 21]. MMS (Modified Morse scale) was used to measure adherence to medication. The reliability of the MMS was tested among similar studies conducted in different regions of Ethiopia [22, 23]. The other variables such as weight, height, blood pressure, and fasting blood sugar were recorded using their standard measurements from the patients. Variables such as duration with diabetes, type of diabetes, presence of complications other than nephropathy, and fasting sugar level were taken from medical history records.

### 2.10. Data Collection Procedure

Data were collected by six trained nurses (BSc) and two supervisors (MSc). Cases and controls were identified from the records of diabetic patients by their identification number. Following the segregation of the cases and controls, data were extracted from each record review card and interviewing the study participants. Moreover, data related to weight, height, and blood pressure were obtained by measuring each of the study participants. Weight was measured in light clothing and without shoes in kilograms (kg) using a calibrated UNICEF Seca digital weighing scale and was checked every six patients by another calibrated UNICEF Seca digital weighing scale [24]. Height was measured using Stadiometer in centimeter (cm) and measurement was checked every six patients by another Stadiometer. While measuring height, study subjects were kept in an erect position such that the back of the head, shoulder blades, buttocks, and heels making contact with the backboard of the Stadiometer [24]. Blood pressure was measured using a mercury sphygmomanometer with a cuff deflation rate of 2 mmHg. The average of two 5 minutes apart measurements of BP from the left arm in sitting position was recorded and each record was checked by another mercury sphygmomanometer [24].

### 2.11. Data Quality Control

A questionnaire prepared in English was translated to the local language (Tigrigna) by an individual who has good ability of the two-language translation. To ensure reliability, it was translated back to English by another individual fluent in both languages. A two-day training was given to data collectors and supervisors in Aksum town. A week prior to the actual data collection, the questionnaire was pretested on 5% of the total sample size in Suhul Hospital. The collected data were reviewed and checked for completeness and consistency by the supervisor and principal investigator on a daily bases during the data collection time.

### 2.12. Data Processing and Analysis

Data were entered into Epi data version 3.1 and analyzed using SPSS version 25 statistical software package. Descriptive statistics including proportion, percentage, ratios, frequency distribution, and mean and standard deviation were determined. Bivariate logistic regression analysis followed by multivariable logistic regression analysis was carried out to determine the association between the independent and outcome variables. Variables with *P* value ≤0.2 in the bivariate logistic regression analysis were entered into multivariable logistic regression. Statistical significance was declared using AOR with odds 95% confidence interval (CI) at *P* value <0.05.

### 2.13. Operational Definitions

  Cases: patients diagnosed with diabetic nephropathy
  Controls: diabetic patients who had no diabetic nephropathy
  Adherence to exercise: respondents who reported that they exercise for >30 minutes per day for at least five times per week
  Adherence to the dietary regimen: this is the strict follow-up of the prescribed dietary regimen for ≥5 days per week
  Adherence to medication: this is the extent of adherence of drug taking behavior of a patient to the agreed-upon recommendations by healthcare provider which was measured with Morisky's 8- item scale where an MMAS-8 score of 6 and above was considered as adherent
  Smoking-related adherence: respondents who reported to have never smoked
  Good glycemic control: a glycemic control was considered to be good when a patient had HbA1c ≤7% and less than 8% for patients with comorbid vascular complications, age greater than 60, and a history of severe hypoglycemia [25]
  Poor glycemic control: a glycemic control was considered to be poor when a patient had HbA1c greater than 7%, and greater than 8% for those patients with comorbid vascular complications, age greater than 60, a history of severe hypoglycemia [25]

## 3. Results

### 3.1. Sociodemographic Characteristics of the Respondents

There were a total of 840 participants (168 cases and 672 controls) with a response rate of 100%. The mean age (±standard deviation) of controls and cases was 45.18 (±8.35) and 62.12 (±13.45) with a minimum and maximum age of 23 and 80 years, respectively. Three hundred sixty-seven (54.6%) controls and eighty-six (51.2%) cases were male. Three hundred seventy-one (55.2%) controls and eighty-three (66.8 %) cases were living in urban areas, and five hundred twenty-one (77.5%) of the controls and one hundred twenty-six (75%) of the cases were married. One hundred fifty-one (22.5%) controls and one hundred twenty-six (17.9%) of the cases completed college and above. Six hundred fifty-nine (98.1%) of the controls and all the cases (100%) were Tegaru, and six hundred forty-six (96.1%) of the controls and all of the cases were Orthodox Christian followers. Around one-fourth (25.75%) of the total controls and forty-five (28.7%) of the cases were private employees (See Table 1).

### 3.2. Health Profile of the Respondents

The mean duration of diabetes mellitus since diagnosis among cases and controls was 16.42 (±4.85) and 6.05 (±2.69), respectively. Five hundred nighty-nine (89.1%) of the controls and one hundred twenty-nine (76.8%) of the cases were members of EDA. Three-fourth (75%) of the controls and one hundred forty-one (83.9%) of the cases had type two DM. Three hundred sixty-one (53.7%) of the controls and one hundred thirty-two (78.6%) of the cases had a medically confirmed comorbidity, of which one hundred thirteen (16.8%) of the controls and ninety-five (56.5%) of the cases had hypertension. One hundred forty-one (21%) of the controls and seventy (41.7%) of the cases had a family history of diabetic mellitus. One-third of the controls (33.3%) and seventy-two (42.9%) of the cases were obese (see Table 2).

### 3.3. Behavioral Factors of the Respondents

Close to three-fourth (74.1%) of the controls and one hundred-two (60.7%) of the cases were found adherent to their antidiabetic medications. But adherence to exercise was found in one hundred sixty-three (24.3%) of the controls and fifty-three (31.5%) of the cases. Two hundred fifty-one (37.4%) of the controls and thirty-five (20.8%) of the cases were adherent to the diabetic diet. More controls (48.2%) than cases (29.2%) had good glycemic control. Similarly, one hundred ninety-one (28.4%) of the controls and fifty-nine (35.1%) of the cases were alcohol consumers. Twenty-one (3.1%) of the controls and five (3%) of cases were smokers (see Table 3).

### 3.4. Determinants of Diabetic Nephropathy

In the bivariable logistic regression analysis, age, residence comorbidity, exercise, having hypertension, duration with diabetes since diagnosis, BMI status, diet, alcohol, family history of diabetic, adherence to medication, glycemic control, and membership of EDA had a *P* value <0.25. However, in the multivariable analysis only, comorbidity [AOR: 4.96 at 95 CI: 1.77–13.87], having hypertension (AOR: 6.33 at 95% CI: 2.51–16.02), poor glycemic control (AOR: 3.27 at 95% CI: 1.31, 8.21), age (AOR: 1.14 at 95%: 1.09–1.19), duration with DM since diagnosis (AOR: 1.83 at 95 CI: 1.62–2.06), and nonadherence to diabetic medication (AOR: 3.31 at 95% CI: 1.34, 8.15), diet (AOR: 5.96 at 95%: 1.92–18.54), and exercise (AOR: 5.60 at 95% CI: 1.94–16.21) were significant predictors of diabetic nephropathy (Table 4).

## 4. Discussion

As the age of diabetic patients increases one year, the chances of diabetic nephropathy increases by 1.14 times, which indicates that the odds of developing diabetic nephropathy increased by 3.74 times for ten years of diabetes age rise. A similar study conducted in Ayder Comprehensive Specialized Hospital, Tigray, showed that age was a determinant factor for diabetic nephropathy [19]. This relationship may be related to the diminished renal function on older DM patients compared to younger ones [26].

The odds of having diabetic nephropathy was 1.83 times higher, with every year increase in duration with diabetes. This indicates having diabetic nephropathy increased by 20.49 times more for five years increments of the duration with diabetes. This fact had been supported by different literature [27–32]. This finding could be explained by the fact that longer duration with DM may increase to have poor glycemic control and comorbidity, which affect renal functions through vascular damage.

The odds of diabetic nephropathy were 5.96 times higher among those nonadherent to diet compared to DM diet adherents. The finding of this study was congruent with a study conducted in Ayder Comprehensive Specialized Hospital, Tigray [33]. Adhering to dietary recommendations can enhance glycemic control and can reduce glycosylated hemoglobin (47) because DM-dietary foods have a low glycemic index which can reduce excess renal function pressure (48, 49).

The chances of diabetic nephropathy among respondents nonadherent to diabetic medications were 3.31 times higher compared to adherents to diabetic medications. This finding is consistent with a study done in Taiwan [34]. This may be due to the fact that nonadherence to diabetic medications contributes to poor glycemic control, which may intensify the gradual deterioration of renal functions, including renal disease in the final stages [35].

Poor glycemic control showed an association with the development of diabetic nephropathy. Similar significant associations were indicated in the findings of other previous studies done in South India, Oman, and westerns countries [29, 30, 36, 37]. This is because poor glycemic control increases the glomerular filtration rate loss and albuminuria [29, 38]. Besides, a high concentration of glucose in meningeal cells causes hypertrophy and increases gene expression and protein secretions which reduces the activity of metalloprotease enzyme which is responsible for the removal of west products [39].

Nonadherence to exercise was strongly significantly associated with diabetic nephropathy (AOR: 3.27 95% CI [1.31, 8.21]). A similar study result was found in Bangalore [32]. The reason may be due to the influences of DM-related exercise on several aspects of diabetic patients including blood glucose absorption, insulin action, and cardiovascular risk factors that preserve renal functions [40].

Having comorbidity also showed a strong significant association with diabetic nephropathy. Similar findings were found in other studies conducted in Bangalore and western countries [32, 37]. This may be due to the reason that comorbidities such as hypertension make arteries around the kidneys narrow and weaken and reduce the blood supply to the kidneys and stop removing waste products from the kidneys that may ultimately damage the kidney itself [41].

## 5. Conclusion

Older age, duration with DM since diagnosis, poor glycemic control, comorbidity, hypertension, and nonadherence to exercise, diet, and medication were found to be the predictors of diabetic nephropathy among DM patients. Adherence to diabetic management (medication, diet, and exercise) should be empowered to achieve glycemic control and to prevent diabetic nephropathy by strengthening information, education, and communication programs. More attention has to be given for older aged diabetic patients, patients with long duration since diagnosis of DM, hypertension, and other comorbidities. Another research should be carried out with another strong design to investigate the determinants of diabetic nephropathy broader social context and in a larger sample size.

## Figures and Tables

**Table 1:** Sociodemographic characteristics of the study participants on follow-up at public hospitals of Tigray, Ethiopia, 2019.

Variables	Category	Controls (*n* = 672)	Cases (*n* = 168)	Total (*n* = 840)
Age	Mean (±SD)	45.18 (8.35)	62.12 (±13.45)	
Sex	Male	367 (54.6%)	86 (51.2%)	453 (53.9%)
	Female	305 (45.4%)	82 (48.8%)	387 (46.1%)
Residence	Urban	371 (55.2%)	83 (49.4%)	454 (54%)
	Rural	301 (44.8%)	85 (50.6%)	386 (46%)
Marital status	Married	521 (77.5%)	126 (75%)	647 (77.0%)
	Single	43 (6.4%)	20 (11.9%)	63 (7.5%)
	Widowed	48 (7.1%)	4 (2.4%)	52 (6.2)
	Divorced	60 (8.9%)	18 (10.7%)	78 (9.3%)
Educational level	Cannot read and write	174 (25.9%)	45 (26.8%)	219 (26.1%)
	Can read and write	89 (13.2%)	0 (0.0%)	89 (10.6)
	Primary y school	144 (21.4%)	56 (33.3%)	200 (23.8%)
	Secondary school	114 (17.0%)	37 (22.0%)	151 (18.0%)
	Colleague and above	151 (22.5%)	30 (17.9%)	181 (21.5%)
Occupation	House wife	171 (25.4%)	32 (19.0%)	203 (24.2%)
	Governmental employee	173 (25.75)	48 (28.6%)	221 (26.3%)
	Private employee	193 (28.7%)	45 (26.8%)	238 (28.3%)
	Daily worker	10 (1.5%)	2 (1.2%)	12 (1.4%)
	Farmer	125 (18.6%)	41 (24.4%)	166 (19.8%)
Ethnicity	Tigray	659 (98.1%)	168 (100.0%)	827 (98.5%)
	Amara	9 (13%)	0 (0.0%)	9 (1.1%)
	Oromo	4 (0.6%)	0 (0.0%)	4 (0.5%)
Religion	Orthodox	646 (96.1%)	168 (100.0%)	814 (96.9%)
	Muslim	26 (3.9%)	0 (0.0%)	26 (3.1%)

**Table 2:** Health profile of the study participants on follow-up at public hospitals of Tigray, Ethiopia, 2019.

Variable	Category	Controls (*n* = 642)	Cases (*n* = 168)	Total (*n* = 840)
Duration with DM	Mean (±SD)	6.05 (±2.69)	16.42 (±4.85)	
Membership EDA	Yes	599 (89.1%)	129 (76.8%)	728 (86.7%)
	No	311 (46.3%)	36 (21.4%)	347 (41.3%)
Medication take	Oral hypoglycemic	527 (78.4%)	138 (82.1%)	665 (79.2%)
	Insulin	37 (5.5%)	3 (1.8%)	40 (4.8%)
	Both	108 (16.1%)	27 (16.1%)	135 (16.1%)
Family history	Yes	141 (21.0%)	70 (41.7%)	211 (25.1%)
	No	531 (79.0%)	98 (58.3%)	629 (74.9%)
Type of DM	Type one	168 (25%)	27 (16.1%)	195 (23.2%)
	Type two	504 (75.0%)	141 (83.9%)	645 (76.8%)
Comorbidity	Yes	361 (53.7%)	132 (78.6%)	493 (58.7%)
	No	311 (46.3%)	36 (21.4%)	347 (41.3%)
Hypertension	Yes	113 (16.8%)	95 (56.5%)	208 (24.8%)
	No	559 (83.2%)	73 (43.5%)	632 (75.2%)
BMI status	Normal	381 (56.7%)	77 (45.8)%	458 (54.5%)
	Overweight	67 (10.0%)	19 (11.3%)	86 (10.2%)
	Obese	224 (33.3%)	72 (42.9%)	296 (35.2%)

**Table 3:** Distribution of behavioral factors among study participants on follow-up at public hospitals of Tigray, Ethiopia 2019.

Variables	Category	Controls (*n* = 672)	Cases (*n* = 168)	Total (*n* = 840)
Adherence medication	Adherent	498 (74.1%)	102 (60.7%)	600 (71.4%)
	Nonadherent	174 (25.9%)	66 (39.3%)	240 (28.6%)
Adherence diet	Adherent	251 (37.4%)	35 (20.8%)	286 (34.0%)
	Nonadherent	421 (62.6%)	133 (79.2%)	554 (66.0%)
Adherence exercise	Adherent	163 (24.3%)	53 (31.5%)	216 (25.7%)
	Nonadherent	509 (75.7%)	115 (68.5%)	624 (74.3%)
Smoker	Yes	21 (3.1%)	5 (3.0%)	26 (3.1%)
	No	651 (96.9%)	163 (97.0)	814 (96.9)
Drinking alcohol	Yes	191 (28.4%)	59 (35.1%)	250 (29.8%)
	No	481 (71.6%)	109 (64.9%)	590 (70.2%)
Glycemic control	Good	324 (48.2%)	49 (29.2%)	373 (44.4%)
	Poor	348 (51.8%)	119 (70.8%)	467 (55.6%)

**Table 4:** Determinants of diabetic nephropathy among study participants on follow-up at public hospitals of Tigray region, Ethiopia, 2019.

Variables	Category	Controls (*n* = 672)	Cases (*n* = 168)	COR (95% CI)	AOR (95% CI)
Age	Mean (±SD)	45.18 (8.35)	62.12 (±13.45)	1.19 [1.16, 1.23]	1.14 [1.09, 1.19]^*∗*^
Duration	Mean (±SD)	6.05 (±2.69)	16.42 (±4.85)	1.86 [1.68, 2.06]	1.83 [1.62, 2.06]^*∗*^
Comorbidity	Yes	361 (53.7%)	132 (78.6%)	3.14 [2.12, 4.71]	4.96 [1.77, 13.87]^*∗*^
	No	311 (46.3%)	36 (21.4%)	1	1
Hypertension	Yes	113 (16.8%)	95 (56.5%)	6.44 [4.46, 9.28]	6.33 [2.51, 16.02]^*∗*^
	No	559 (83.2%)	73 (43.5%)	1	1
Adherence medication	Adherent	498 (74.1%)	102 (60.7%)	1	1
	Nonadherent	174 (25.9%)	66 (39.3%)	1.85 [1.29, 2.64]	3.31 [1.34, 8.15]^*∗*^
Adherence diet	Adherent	251 (37.4%)	35 (20.8%)	1	1
	Nonadherent	421 (62.6%)	133 (79.2%)	2.26 [1.51, 3.39]	5.96 [1.92, 18.54]^*∗*^
Adherence to exercise	Adherent	163 (24.3%)	53 (31.5%)		1
	Nonadherent	509 (75.7%)	115 (68.5%)		5.60 [1.94, 16.21]^*∗*^
Glycemic control	Good	324 (48.2%)	49 (29.2%)	1	1
	Poor	348 (51.8%)	119 (70.8%)	2.26 [1.57, 3.26]	3.27 [1.31, 8.21]^*∗*^

^*∗*^The determinants of diabetic nephropathy at *P* value <0.005.

## Data Availability

The data sets used and analyzed during the current study are available from the corresponding author upon request.

## Abbreviations

AOR: Adjusted odds ratio
COR: Crude odds ratio
CVD: Cardiovascular disease
DM: Diabetes Mellitus
FBS: Fasting blood sugar
HbA1c: Hemoglobin A1c
IEC: Information education and communication
IRB: Institutional review board
MMS: Modified Morse scale
SMBG: Self-monitoring of blood glucose
SDCA: Summary of diabetes care activity
UNICEF: United Nations International Child Fund.
